# PD-1/PD-L1 expression and tumor-infiltrating lymphocytes are prognostically favorable in advanced high-grade serous ovarian carcinoma

**DOI:** 10.1007/s00428-020-02751-6

**Published:** 2020-01-24

**Authors:** Laura Martin de la Fuente, Sofia Westbom-Fremer, Nicolai Skovbjerg Arildsen, Linda Hartman, Susanne Malander, Päivi Kannisto, Anna Måsbäck, Ingrid Hedenfalk

**Affiliations:** 1 Department of Clinical Sciences, Division of Oncology and Pathology, Lund University and Skåne University Hospital, Lund, Sweden; 2 Department of Surgical Pathology, Division of Laboratory Medicine, Skåne University Hospital, Lund, Sweden; 3 Department of Obstetrics and Gynaecology Lund, Skåne University Hospital, Lund University, Lund, Sweden

**Keywords:** PD-1/PD-L1 pathway, Tumor-infiltrating lymphocytes, Macrophages, Prognostic marker

## Abstract

The response rate to checkpoint inhibitors for women with high-grade serous carcinoma of the ovary, fallopian tube, and peritoneum (HGSC) is modest, and development of predictive biomarkers is needed. The main focus has been on tumor cell PD-L1 expression, but its assessment alone is insufficient for patient selection in most malignancies. We mapped the presence of macrophages (CD68 and CD163) and lymphocytes (CD3) located within the tumor epithelium, the cell type–specific expression of PD-L1 and PD-1, and their impact on 5-year overall survival (OS) in a consecutive cohort of 130 women diagnosed with advanced HGSC between 2011 and 2015. PD-L1 was expressed mainly by macrophages (not by tumor cells) and PD-1 by lymphocytes. Women with higher CD3, PD-L1, and PD-1 expression had improved OS (*P* = 0.03, *P* = 0.007, and *P* = 0.02, respectively). In the external data set (203 women), high expression of *CD274* (encoding PD-L1) was associated with improved OS (*P* = 0.03), in accordance with our results. Furthermore, higher CD163 expression was associated with better outcome in women with no residual tumor after primary surgery (*P* = 0.02). Thus, women with greater lymphocyte tumor infiltration had better outcome and PD-L1/PD-1 expression, regardless of PD-1/PD-L1 being markers for immune suppressive pathways, conferred a survival benefit in our cohort. Our results highlight that tumor immunity may be harnessed in subsets of HGSC.

**Electronic supplementary material:**

The online version of this article (10.1007/s00428-020-02751-6) contains supplementary material, which is available to authorized users.

## Introduction

High-grade serous carcinoma of the ovary, fallopian tube, and peritoneum (HGSC) is the most common and lethal subtype of epithelial ovarian carcinoma [1]. Due to the lack of symptoms, it typically presents at an advanced stage. Cytoreductive surgery is the most important treatment affecting outcome, and patients with no macroscopic residual tumor after primary surgery have a survival benefit [2]. Surgery is followed by platinum-based adjuvant chemotherapy. Despite a majority of women with advanced HGSC initially responding to treatment, many suffer relapses and the cancer cells have then often developed resistance or are less sensitive to chemotherapy. Thus, advanced stage at diagnosis and a high rate of relapses are the main reasons for the poor prognosis of this disease. Although no great improvements in outcome have been made over the past decades, the recent introduction of poly (ADP-ribose) polymerase (PARP) inhibitors into the clinical practice seems promising in transforming survival prospects for women with advanced HGSC [3].

The introduction of checkpoint inhibitor-based antibodies directed at CTLA-4, PD-1, and PD-L1 receptors has improved survival for many cancer patients. In particular, in advanced malignant melanoma, lung cancer and bladder cancer clinical trials have shown increased overall survival (OS) and longtime survivors [4–6]. To date, the response rate to checkpoint inhibitors for patients with HGSC seems to be modest [7, 8]. However, there is hope for increased response rates through patient selection and combination of therapies. For example, there is emerging preclinical data suggesting that the patient population that responds to PARP inhibition and PD-1/PD-L1 antibodies may significantly overlap [9], and it is hypothesized that increased DNA damage by PARP inhibition will increase the number of tumor neoantigens, creating a more antigenic environment in which to stimulate the immune microenvironment [10]. Thus, development of predictive biomarkers is needed to identify the subset of patients who will benefit from treatment and to minimize the risk of toxicities. The main focus to date has been on tumor cell PD-L1 expression, but its assessment alone is insufficient for patient selection in most malignancies [11].

The survival advantage of high numbers of tumor-infiltrating lymphocytes in HGSC has been shown in several studies [12, 13]. Furthermore, global gene expression analyses have identified an immunoreactive molecular subtype [14, 15], and showed its value as a predictor of improved survival compared with the other molecular subtypes [16]. However, the prognostic value of PD-1 and PD-L1 in HGSC has been studied with ambiguous results [17–20]. Mapping the expression of PD-1/PD-L1 and immune cells in HGSC is clinically relevant because in addition to its prognostic value, it may provide important information for further study of their potential to predict treatment response to immunotherapy. Furthermore, a negative effect of CD163+ tumor-associated macrophages on survival was reported in a meta-analysis of patients with ovarian carcinoma [21]. Therefore, we characterized the macrophage population by evaluating CD163 in our cohort, a marker for alternatively activated macrophages (M2) considered to promote tumor progression.

Thus, in this study, we mapped the presence of macrophages and lymphocytes located within the tumor epithelium, the cell type–specific expression of PD-L1 and PD-1 and their impact on prognosis in a well-characterized, contemporary and consecutive cohort of 130 women diagnosed with advanced HGSC.

## Methods

This study followed the REMARK (Reporting Recommendations for Tumor Marker Prognostic Studies) guidelines [22].

### Patients

Ethical approval for this study was granted by the Ethics Committee at Lund University, Sweden, waiving the requirement for informed consent. A total of 156 consecutive cases of HGSC were selected at the Gynecology Department in the southern Swedish healthcare region between 2011 and 2015. All cases were reviewed by a gynecologic pathologist according to the World Health Organization Classification 2014 [23] (excluded patients shown in Supplementary Fig. 1) and staged according to the International Federation of Gynecology and Obstetrics criteria [24]. All tumor samples were collected at primary surgery or diagnostic biopsy prior to chemotherapy administration. Eleven women with stage I and II disease were excluded from the analysis as we were not able to find regression model fitting all stages and due to their remarked difference in prognosis. Thus, 130 women with stage III and IV disease were included in the analysis. With the exception of three patients who underwent bowel obstruction surgery only, all patients underwent primary cytoreductive surgery (two patients delayed primary and the rest upfront primary). The complete resection rate (no macroscopic tumor) for upfront and delayed primary surgery was 75/127 (59%). Four cases, where upfront primary cytoreductive surgery failed, were redirected to neoadjuvant chemotherapy, and of the two that could undergo interval surgery, none achieved complete resection. Platinum-based chemotherapy was administered to all but four of the 130 patients, and three patients died after only one chemotherapy cycle had been administered. Of the 123 patients who completed chemotherapy, 87 (71%) were evaluated as complete response, 29 (24%) as partial response, one as stable disease and six as progressive disease, by the end of treatment. Platinum resistance was defined according to the consensus achieved by the Gynecologic Cancer InterGroup (Vancouver, June 2010) [25], and data are presented in Table 1. Sixteen patients with residual tumor after primary surgery received bevacizumab together with the platinum-based regime. Last follow-up date for all patients was December 2018. Median follow-up time for the patients included was 39 months (0.3–89.0) (events and more detailed survival data in Table 1).

**Table 1:** PD-1, PD-L1, CD3, CD68, and CD163 expression and clinical parameters

			*N* (%)	PD-1 low^a^ *N* (%)		PD-1 high^a^ *N* (%)	*P* value	PD-L1 low^a^ *N* (%)	PD-L1 high^a^ *N* (%)	*P* value
Advanced HGSC			130	91		39		104	26	
Age										
Mean Range			67 43–86	68 43–86		65 45–85	0.5^c^	68 43–86	63 51–85	0.2^c^
Residual tumor										
No Yes			75 (58) 55 (42)	50 (55) 41 (45)		25 (64) 14 (36)	0.3^d^	57 (55) 47 (45)	18 (69) 8 (31)	0.2^d^
Stage										
III IV			99 (76) 31 (24)	67 (74) 24 (26)		32 (82) 7 (18)	0.3^d^	77 (74) 27 (26)	22 (85) 4 (15)	0.3^d^
PFI										
12 months 6–12 months < 6 months No platinum			64 (52) 29 (24) 30 (24) 7	37 (44) 25 (29) 23 (27)		27 (71) 4 (11) 7 (18)	*0.009*^*e*^	49 (50) 24 (25) 25 (25)	15 (60) 5 (20) 5 (20)	0.3^e^
5-Year OS										
Events/person years 5-Year OS (%)			85^b^/388 29.5	67/253 21.7		18/135 49.3		74/298 23.6	11/90 56.4	
5-Year PFS										
Events/person years 5-Year PFS (%)			107/257 16.5	82/155 9.4		25/102 33		89/196 14	18/61 26	
		CD3 low *N* (%)	CD3 high *N* (%)	*P* value	CD68 low *N* (%)	CD68 high *N* (%)	*P* value	CD163 low *N* (%)	CD163 high *N* (%)	*P* value
Advanced HGSC		87	43		60	70		79	51	
Age										
Median Range		68 43–86	65 45–85	0.08^c^	68 45–86	65 45–85	*0.03*^*c*^	68 45–86	66 43–80	0.1^c^
Residual tumor										
No Yes		52 (60) 35 (40)	23 (53) 20 (47)	0.5^d^	38 (63) 22 (37)	37 (53) 33 (47)	0.2^d^	47 (49.5) 32 (40.5)	28 (55) 23 (45)	0.6^d^
Stage										
III IV		65 (75) 22 (25)	34 (79) 9 (21)	0.6^d^	47 (78) 13 (22)	52 (74) 18 (26)	0.6^d^	60 (76) 19 (24)	39 (76.5) 12 (23.5)	0.9^d^
PFI										
> 12 months 6–12 months < 6 months		39 (47) 21 (26) 22 (27)	25 (61) 8 (19.5) 8 (19.5)	0.6^e^	28 (50) 14 (25) 14 (25)	36 (54) 15 (22) 16 (24)	0.6^e^	37 (49) 15 (20) 23 (31)	27 (56) 14 (29) 7 (15)	0.3^e^
5-Year OS										
Events/person years 5-Year OS (%)		63/245 24.2	22/143 41.1		44/172 24.4	41/216 34.4		56/226 24.1	29/162 38.1	
5-Year PFS										
Events/person years 5-Year PFS (%)		78/156 9.2	29/101 32.6		54/108 8.7	53/149 23.1		68/143 13.9	39/114 21.2	

Values in italics are statistically significant (*P* < 0.05)

*PFI* platinum-free interval, *PFS* progression-free survival defined as the time interval between date of diagnosis and the date of disease recurrence (pathology report or radiology) or death, whichever occurred first

^a^PD-1 expression in intra-epithelial lymphocytes and PD-L1 expression in intra-epithelial macrophages

^b^Of the patients who died within 5 years after diagnosis, all but two of 85 died of causes related to HGSC

^c^*t* test

^d^Chi^2^ test

^e^Mann-Whitney *U* test

### Tissue microarray construction and immunohistochemistry

Viable tumor areas from multiple sites were selected from formalin-fixed paraffin-embedded tissue blocks: four cores from the adnexa (two different blocks), two cores from lymph node metastases (if present), and two or four cores from peritoneal metastases. Thus, 6–8 1-mm core needle biopsies from multiple sites were available from each patient in most cases (of 130 women, 19 cases had 4 cores, 68 cases 6 cores, and 43 cases 8 cores). Sections, 3–4 mm in thickness, were deparaffinized, rehydrated, and stained. A summary of antibodies and immunohistochemistry procedures used is provided in Table 2. The sections were incubated with primary antibody (detailed incubation conditions in Table 2). The visualization systems applied were EnVision FLEX (Agilent Dako) for the Dako Autostainer platform and ultraView Universal Detection Kit for the Ventana platform. Placenta and macrophages in tonsil were used as a positive control for PD-L1 (high and low expression, respectively). Macrophages and lymphocytes, and epithelial cells in tonsil, were positive and negative controls, respectively, for CD68, CD163, CD3, and PD-1.

**Table 2:** Summary of antibodies and immunohistochemistry procedures

Antigen	Clone	Cat. no.	Supplier	Dilution	Platform	Ag retrieval (pH)	Ab incubation (min/°C or RT)
PD-L1	22C3	M3653	Agilent Dako	1:50	Dako Autostainer	DT 1699 (6)	30/RT
PD-1	NAT105	315M	Cell Marque (Sigma)	1:100	Dako Autostainer	DT 1699 (6)	30/RT
CD68	PG-M1	M0876	Agilent Dako	1:100	Dako Autostainer	DT 2367 (9)	30/RT
CD163	MRQ-26	760-4437	Ventana	RTU	Ventana Benchmark Ultra	CC1 (8.5)	32/36
CD3	Poly	A0452	Agilent Dako	1:200	Dako Autostainer	DT 2367 (9)	30/RT
Large sections							
PD-L1	22C3	M365529	Agilent Dako	1:40	Ventana Benchmark Ultra	CC1 (8.5)	64/36
PD-1	NAT105	Ab52587	Abcam	1:50	Ventana Benchmark Ultra	CC1 (8.5)	32/36
PD-1	NAT105	315M	Cell Marque (Sigma)	1:100	Dako Autostainer	DT 1699 (6)	30/RT
CD68	PG-M1	M0786	Dako	1:100	Ventana Benchmark Ultra	CC1 (8.5)	32/36
CD3	2GV6	760-4341	Ventana	RTU	Ventana Benchmark Ultra	CC1 (8.5)	32/36

*RT* room temperature, *RTU* ready to use, *DT 1699* Dako Target retrieval solution 1699, *DT 2367* Dako Target retrieval solution 2367, *CC1* cell conditioning 1

### Scoring

**Fig. 1:**
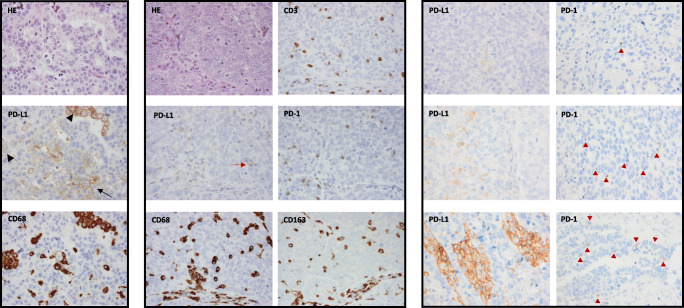
The macrophage marker CD68 facilitated the distinction between macrophages and cancer cells when evaluating PD-L1 expression (left panel). Pictures of corresponding tumor areas on consecutive tissue microarray sections. Arrowheads show PD-L1 expression in macrophages and arrow PD-L1 expression by cancer cells. Example of a positive case for all immunohistochemical staining (middle panel). The red arrow shows a PD-L1 positive macrophage. Stroma seen at the bottom of the pictures excluded from scoring. Examples of areas rich in PD-L1 expression in intra-epithelial macrophages and PD-1 expression in intra-epithelial lymphocytes from cores with scores < 1%, 1–4%, and ≥ 5% (from top to bottom) (right panel). The red arrowheads show PD-1 positive lymphocytes. Magnification, ×40

Hematoxylin and eosin, PD-L1, CD68, CD3, PD-1, and CD163 were stained on consecutive sections enabling the evaluation of corresponding tumor areas. We scored lymphocytes located within the tumor epithelium, and only intra-epithelial and luminal macrophages were evaluated. All stained slides, except PD-L1, were evaluated by two investigators (LMF, SWF), and discordant cases were discussed until consensus was achieved. PD-L1 evaluation was performed by a pathologist (SWF) with experience in PD-L1 scoring of lung cancer in the clinical setting. Scoring was performed blinded from clinical data. The macrophage marker CD68 facilitated the distinction between macrophages and cancer cells when evaluating PD-L1 expression (see Fig. 1). We determined the average (0%, < 1%, 1–4%, ≥ 5% for PD-L1 and PD-1 and 0%, < 1%, 1%, 2–4%, ≥ 5% for CD3, CD68, and CD163) of the total cell amount in each core, excluding areas with stroma, acute inflammation, and necrosis. Examples of score intervals for PD-L1 and PD-1 are presented in Fig. 1. Further, we stained and evaluated 13 cases of paired tissue microarray and whole sections for comparison.

### In silico validation of CD274 (PD-L1) and CD3G (CD3) mRNA expression

An independent public gene expression data set consisting of 285 high-grade serous and endometrioid, borderline as well as low-grade serous and endometrioid ovarian tumors, fallopian tube, and primary peritoneal cancers was downloaded from Gene Expression Omnibus (GSE9891) [14]. We selected 203 cases with high-grade serous histology and studied the relationship between expression of *CD274* (probe 227458_at, encoding PD-L1) and *CD3G* (probe 206804_at, encoding CD3) and 5-year OS. We compared high versus low expression using the median mRNA expression level as cutoff.

### Statistical analyses

The prognostic value was investigated using 5-year OS as endpoint, defined as the time interval between date of diagnosis and death.

Statistical analyses were performed with the Statistical Package for the Social Sciences, Windows version 25. Survival analyses were performed using the Kaplan-Meier method, and differences between groups were tested using the log-rank test. The effect of the expression of the immune markers on OS was expressed using hazard ratios (HRs) with 95% confidence interval (CI), estimated using univariable and multivariable Cox regression. The multivariable analysis adjusted for clinical factors known to influence HGSC survival, age at diagnosis (≥ 70 vs. < 70), stage (IV vs. III), and residual tumor following primary cytoreductive surgery (macroscopic residual tumor vs. no) [1, 26, 27], were analyzed as binary factors. All *P* values are two sided. The three patients who underwent bowel obstruction surgery only were considered as having macroscopic residual tumor for analyses.

In the external data set, statistical analyses were performed in R version 3.3.3. Associations between *CD3G* and *CD274* mRNA levels and 5-year OS were assessed using the Kaplan-Meier method, and HRs with 95% confidence interval were calculated in univariable analysis using Cox proportional hazard regression (R “survival” package version 2.41-3).

## Results

We found significant positive associations between all markers studied, except PD-1 and CD163 (chi^2^, *P* = 0.06). Strong positive associations were observed between PD-L1, PD-1, and CD3 (chi^2^, *P* < 0.001); PD-1, CD3, and CD68 (chi^2^, *P* < 0.001); and PD-L1, CD3, CD163, and CD68 (chi^2^, *P* < 0.01). Associations between expression of each marker and clinical parameters are shown in Table 1.

### Patterns and prognostic effects of PD-1 and PD-L1 expression

**Fig. 2:**
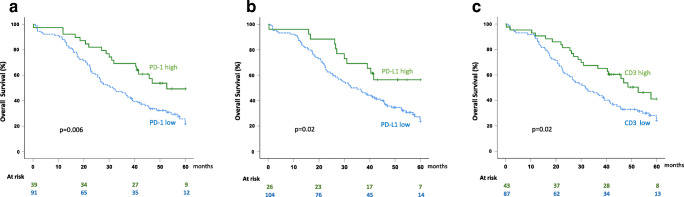
Association between OS and the expression of PD-1 in lymphocytes, PD-L1 in macrophages, and CD3 expression, within the tumor epithelium. **a** Patients with high PD-1 expression had longer survival compared with patients with low PD-1 expression. **b** Patients with high PD-L1 expression had longer survival compared with patients with low PD-L1 expression. **c** Patients with high CD3 expression had longer survival compared with patients with low CD3 expression. *P* values were calculated using the log-rank test

PD-1 was almost exclusively expressed by lymphocytes (Fig. 1). We observed both partial and complete, as well as weak and moderate, membranous staining. Patients with ≥ 50% cores with PD-1 expression ≥ 1% (39/130), considered to have high expression, had longer OS (*P =* 0.007; Fig. 2a and Table 3), even when adjusting for well-known prognostic factors (Table 4). Interestingly, high PD-1 expression was associated with a stronger survival benefit when the analysis was restricted to the 43 cases with high CD3 expression (31 cases PD-1 high vs. 12 cases PD-1 low, HR 0.33 [0.14–0.77], *P =* 0.01).

**Table 3:** Univariable analyses of overall survival

		5-Year OS univariable Cox		
		*n* (events)	HR (95% CI)	*P*
CD3^a^	Low	87 (63)	1	
	High	43 (22)	0.58 (0.35–0.94)	*0.03*
PD-1^a^	Low	91 (67)	1	
	High	39 (18)	0.49 (0.29–0.82)	*0.007*
PD-L1^a^	Low	104 (74)	1	
	High	26 (11)	0.47 (0.25–0.89)	*0.02*
Age at diagnosis	< 70	85 (47)	1	
	≥ 70	45 (38)	2.5 (1.6–3.8)	*< 0.001*
Stage	III	99 (57)	1	
	IV	31 (28)	2.9 (1.8–4.5)	*< 0.001*
Residual tumor	No	75 (42)	1	
	Yes	55 (43)	2.0 (1.3–3.1)	*0.001*

Values in italics are statistically significant (*P* < 0.05)

^a^Intra-epithelial CD3 expression, PD-1 expression in intra-epithelial lymphocytes, and PD-L1 expression in intra-epithelial macrophages

**Table 4:** Multivariable analyses of overall survival

		5-Year OS multivariable Cox	
		HR (95% CI)	*P*
CD3^a^	High vs. low	0.60 (0.37–0.98)	*0.04*
Age at diagnosis	≥ 70 vs. < 70	2.3 (1.5–3.6)	*< 0.001*
Stage	IV vs. III	2.8 (1.8–4.5)	*< 0.001*
Residual tumor	Yes vs. no	1.8 (1.2–2.7)	*0.01*
PD-1^a^	High vs. low	0.55 (0.32–0.94)	*0.03*
Age at diagnosis	≥ 70 vs. < 70	2.3 (1.5–3.5)	*< 0.001*
Stage	IV vs. III	2.6 (1.6–4.2)	*< 0.001*
Residual tumor	Yes vs. no	1.8 (1.2–2.8)	*0.008*
PD-L1^a^	High vs. low	0.62 (0.32–1.2)	0.1
Age at diagnosis	≥ 70 vs. < 70	2.3 (1.5–3.6)	*< 0.001*
Stage	IV vs. III	2.8 (1.5–3.6)	*< 0.001*
Residual tumor	Yes vs. no	1.7 (1.1–2.6)	*0.02*

Multivariable analysis of each immune marker including well-known prognostic factors above. Values in italics are statistically significant (*P* < 0.05)

^a^Intra-epithelial CD3 expression, PD-1 expression in intra-epithelial lymphocytes, and PD-L1 expression in intra-epithelial macrophages

PD-L1 was expressed mainly by macrophages, and to a far lesser extent by tumor cells and lymphocytes (Fig. 1). We evaluated the membranous expression that was predominantly partial and weak, but also observed granular cytoplasmic expression. Using the same cutoff as for PD-1 (≥ 50% cores with ≥ 1% PD-L1 expressing macrophages), we identified only 15/130 patients as positive. The survival benefit of this small group was high and statistically significant but uncertain (HR 0.20 [0.07–0.66], *P =* 0.007). Thus, we considered cases with ≥ 2 cores with ≥ 1% PD-L1 expressing macrophages as positive (26/130) and found a significant association with improved OS also within this group (*P =* 0.02; Fig. 2b and Table 3). Because of scarce PD-L1 expression in tumor cells and lymphocytes, no relevant cutoff could be determined, precluding further analyses.

Furthermore, we explored the survival benefit of the subgroup with higher expression of both PD-1 and PD-L1 and found lower hazards of death compared with each marker alone (19 cases PD-1/PD-L1 high vs. 84 cases PD-1/PD-L1 low, HR 0.36 [0.17–0.79], *P =* 0.01, Supplementary Table 1).

### Lymphocytes and macrophages in HGSC

Patients with ≥ 50% cores with ≥ 2% lymphocytes (CD3 high, 43/130) had longer OS compared with patients with lower expression (*P =* 0.03; Fig. 2c and Table 3), even when adjusting for well-known prognostic factors (*P =* 0.04; Table 4).

No significant OS difference between patients with ≥ 50% cores with ≥ 2% macrophages (CD68 high, 70/130) and patients with lower CD68 expression (60/130) was observed (HR 0.74 [0.48–1.1], *P =* 0.2). Furthermore, we did not find any difference in OS between patients with ≥ 50% cores with CD163 expression ≥ 2% (CD163 high, 51/130) and patients with lower CD163 expression (79/130, HR 0.70 [0.44–1.1], *P =* 0.1). However, in the subgroup of patients with no macroscopic residual tumor, higher CD163 expression was associated with better outcome (*P =* 0.02, Supplementary Table 2).

### Whole tissue sections

We observed concordance between evaluations performed on tissue microarray and whole tissue sections in the majority of the 17 pairs investigated (13 cases, four of which with paired samples from adnexa and metastatic site). Comparing the average staining of the cores and whole tissue sections, the highest concordance was observed for PD-1, where only 1/17 pairs was discordant. Regarding PD-L1 in macrophages, scoring on tissue microarray underestimated whole tissue section scoring in 3/17 pairs and for CD3, and 2/17 pairs were discordant.

Of note, tissue microarray and whole section immunostainings were performed on the Dako Autostainer and Ventana system, respectively, which did not seem to affect the performance of the antibodies. PD-1 immunostaining on whole sections was discordant on the Ventana system, and therefore, we performed the comparison on the same platform as the tissue microarray, the Dako Autostainer.

### High CD274 mRNA expression linked with survival benefit

In the external data set, high expression of *CD274* (encoding PD-L1) was associated with improved OS among HGSC patients (*P* = 0.03; Supplementary Fig. 2). However, no significant associations between *CD3G* (encoding CD3) and OS were found.

## Discussion

In this study, we found that PD-L1 was expressed mainly by macrophages, which has previously been reported [19, 28, 29] and not by tumor cells as some previous studies suggest [17, 18, 20, 30]. Remarkably, macrophage staining, which was necessary for the correct mapping of PD-L1 expression in macrophages vs. tumor cells and revealed the predominance of expression on macrophages, was not performed in the previous studies where PD-L1 expression in macrophages was not described. Moreover, the specificity and sensitivity of anti-PD-L1 antibodies have been debated, and based on results from The Blueprint Project [31] and Brunnström et al. [32], we decided to use the FDA-approved 22C3 clone in the present study.

In our cohort of 130 consecutive cases of advanced HGSC, higher expression of intra-epithelial lymphocytes (CD3), PD-1 (in lymphocytes), and PD-L1 (in macrophages) was a predictor of better outcome with similar hazards of death, even after adjusting for age at diagnosis, stage, and residual tumor after primary surgery. In agreement with our results, previous studies have reported the significantly decreased risk of death in patients with higher intra-epithelial CD3 expression (HR 0.50 [0.36–0.69] and HR 0.45 [0.34–0.58] in two different meta-analyses) [12, 13]. Regarding PD-1, two previous studies have showed a survival benefit for patients with ovarian carcinoma whose tumors express PD-1 [18, 33]. However, in one of the studies, PD-1 expression was described in tumor cells in a majority of the cases (151/172) in addition to lymphocytes [18]. In our study, we did not observe any PD-1 staining in tumor cells, consistent with one other previous report [33]. In fact, the study reporting PD-1 expression in tumor cells used different PD-1 antibodies to stain tissue microarray and whole sections, and importantly, no PD-1-positive cancer cells were observed in whole sections.

Higher PD-L1 expression predicts inferior survival in several cancer forms [34], supporting the theory of tumor cells upregulating PD-L1 in order to suppress T cells, thereby promoting tumor growth. In contrast, previous studies in HGSC, in addition to our cohort and in silico validation presented herein, have shown a survival benefit of high PD-L1 expression in macrophages [19, 28, 29]. Interestingly, high PD-1 expression was associated with an even stronger survival benefit when the analysis was restricted to cases with high CD3 expression. Thus, despite the PD-1/PD-L1 pathway being a negative regulator of T cell activation, women which tumors had a higher PD-1 expression in intra-epithelial lymphocytes and a higher PD-L1 expression in intra-epithelial macrophages had longer survival. Indeed, a previous study referred to adaptive immune resistance, wherein activated T cells may trigger negative feedback mechanisms, resulting in an immunological stalemate [19]. The sparse expression of PD-L1 in HGSC tumor cells, in contrast to other malignancies [31], remains an unresolved issue. Nevertheless, the observed survival benefit suggests that the potential of tumor immunity may be harnessed in subsets of HGSC.

We did not observe a prognostic value of intra-epithelial macrophages (CD68) in the present study. Only two previous studies—smaller and including mixed histological types—were found, making it difficult to compare with our study [35, 36]. Similarly, some smaller studies with mixed histological types, evaluating CD163 expressing macrophages in ovarian carcinoma, have been published. Apparently, the results of a previous study that showed a survival benefit for patients whose tumors displayed low CD163 expression in multivariable analysis [37] are in conflict with our results. However, in this study, peritumoral stroma and not intra-epithelial CD163 expression were evaluated. Another study did not report any difference in prognosis between groups with high vs. low intra-epithelial CD163 expression [38], which is in agreement with our results. However, higher CD163 expression was associated with better outcome in the subgroup of patients with no macroscopic tumor after surgery in our cohort. This result is apparently in conflict with the theory that M2 macrophages promote tumor progression, thereby negatively affecting survival [39]. Of note, the macrophage classification in classical versus alternative activated represents a simplification of the heterogeneous macrophage population in tumors [39], and a more detailed characterization of macrophages in HGSC may be required.

Although some previous studies have investigated the prognostic value of PD-L1 and PD-1 in HGSC, we found ambiguous issues that we addressed in the present study. According to our results, the main cells expressing PD-L1 are macrophages (and not tumor cells) and that PD-1 is almost exclusively expressed by lymphocytes (and not by tumor cells). Most previous studies include smaller cohorts and, in some cases, not consecutively collected cohorts with a selected material concerning residual disease [19] or stage [30]. Further, this is the first study reporting on the prognostic value of CD68 and CD163 in a pure advanced HGSC cohort, as previous smaller studies included mixed histologies which differ greatly in clinical presentation and prognosis. Other strengths of this study include the use of a contemporary and comprehensive tissue microarray, as well as validation using tissue whole sections. Given the number of cores per case and the fact that the tissue microarray includes cores from both primary and metastatic sites, our tissue microarray outperforms previous ovarian carcinoma tissue microarray cohorts. Furthermore, the PD-L1 scoring was performed by a pathologist with experience from PD-L1 scoring of lung cancer in the clinical setting.

Some of the limitations include that only 25 patients were tested for *BRCA1/2* mutations in our cohort, precluding the possibility of performing potentially interesting survival analyses in these subgroups. However and interestingly, we noted a strong positive correlation between the expression of the immune marker that best predicted prognosis, PD-1, and platinum sensitivity. The group of platinum sensitive tumors is enriched with tumors showing homologous recombination deficiency [40], and this may imply a relationship between tumor immunogenicity and homologous recombination deficiency, as recently suggested [10].

In conclusion, we corroborate that PD-L1 is primarily expressed by macrophages and found that higher expression of lymphocytes, PD-1 in lymphocytes, and PD-L1 in macrophages within the tumor epithelium confers a significant survival advantage in advanced HGSC.

## Electronic supplementary material

ESM 1 (DOCX 15 kb)

ESM 2 (PDF 111 kb)
