# Supplementary material for: PD-1/PD-L1 expression and tumor-infiltrating lymphocytes are prognostically favorable in advanced high-grade serous ovarian carcinoma
Source: Virchows Arch. 2020 Jan 24;477(1):83–91. doi: 10.1007/s00428-020-02751-6 (PMC7320055; doi:10.1007/s00428-020-02751-6)
Supplement: Supplementary file 1 — (DOCX 15 kb) [file 428_2020_2751_MOESM1_ESM.docx]

**Supplementary Table 1. Univariable and multivariable analyses of overall survival of PD-1/PD-L1 combinations**

|  | | **5-Year OS Univariable Cox** | | |
| --- | --- | --- | --- | --- |
|  |  | n (events) | HR (95% CI) | *P* |
| **PD-1/PD-L1*** **combination** | **PD1 low/PDL1 low** | 84 (63) | 1 |  |
|  | PD1 low/PDL1 high | 7 (4) | 0.61 (0.22-1.7) | 0.3 |
|  | PD1 high/ PDL1 low | 20 (11) | 0.57 (0.30-1.1) | 0.09 |
|  | **PD1 high/ PDL1 high** | 19 (7) | 0.36 (0.17-0.79) | **0.01** |
|  | | **5-Year OS Multivariable Cox** | | |
|  |  | n (events) | HR (95% CI) | *P* |
| **PD-1/PD-L1*** **combination** | **PD1 low/PDL1 low** | 84 (63) | 1 |  |
|  | PD1 low/PDL1 high | 7 (4) | 0.85 (0.30-2.4) | 0.8 |
|  | PD1 high/ PDL1 low | 20 (11) | 0.61 (0.31-1.2) | 0.1 |
|  | **PD1 high/ PDL1 high** | 19 (7) | 0.47 (0.21-1.1) | 0.06 |
| **Age at diagnosis** | ≥70 vs. <70 | 130 (85) | 2.2 (1.4-3.5) | **<0.001** |
| **Stage** | IV vs. III | 130 (85) | 2.6 (1.6-4.3) | **<0.001** |
| **Residual tumor** | Yes vs. No | 130 (85) | 1.8 (1.1-2.7) | **0.01** |

*PD-1 expression in intra-epithelial lymphocytes and PD-L1 expression in intra-epithelial macrophages.

**HR**, hazard ratio. **CI**, confidence interval. Values in bold are statistically significant (*P*<0.05).

**Supplementary Table 2. Univariable analyses of overall survival of CD163 by subgroups no residual tumor/residual tumor**

|  | | | **5-Year OS Univariable Cox** | | |
| --- | --- | --- | --- | --- | --- |
|  |  |  | n (events) | HR (95% CI) | *P* |
| No residual tumor | **CD163*** | Low | 47 (31) | 1 |  |
|  |  | High | 28 (11) | 0.44 (0.22-0.88) | **0.02** |
| Residual tumor |  | Low | 32 (25) | 1 |  |
|  |  | High | 23 (18) | 1.1 (0.58-2.0) | 0.8 |

* Intra-epithelial CD163 expression.

**HR**, hazard ratio. **CI**, confidence interval. Values in bold are statistically significant (*P*<0.05).
