# Supplementary material for: PD-1/PD-L1 expression and tumor-infiltrating lymphocytes are prognostically favorable in advanced high-grade serous ovarian carcinoma
Source: Virchows Arch. 2020 Jan 24;477(1):83–91. doi: 10.1007/s00428-020-02751-6 (PMC7320055; doi:10.1007/s00428-020-02751-6)
Supplement: Supplementary file 2 — (PDF 111 kb) [file 428_2020_2751_MOESM2_ESM.pdf]

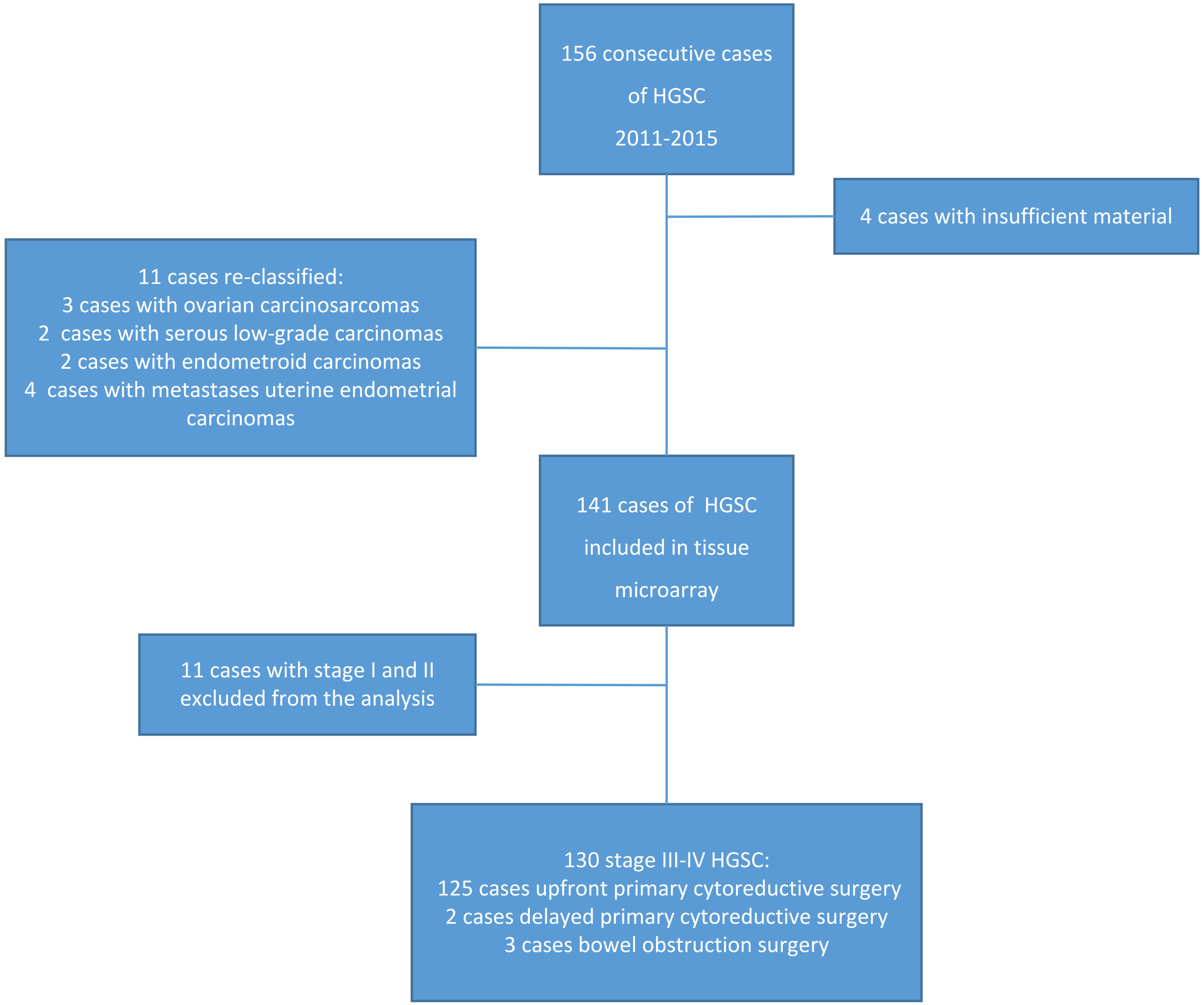

Supplementary Figure 1. Flowchart of patient inclusion. HGSC, high grade serous ovarian carcinoma.

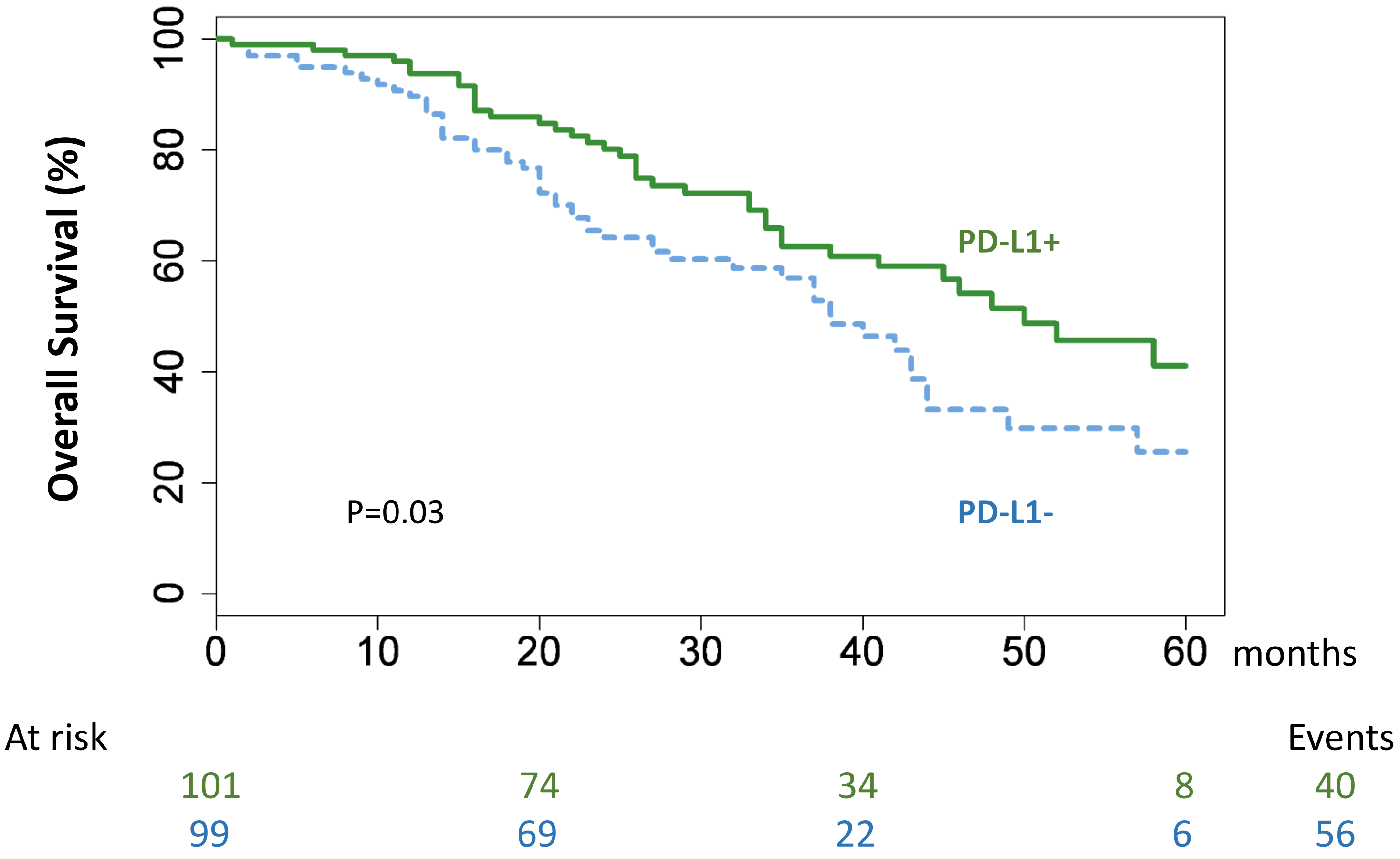

Supplementary Figure 2. Kaplan-Meier analysis comparing high versus low *CD274* mRNA expression groups using the median mRNA expression level as cut-off. Of the 203 cases of ovarian high-grade serous carcinomas 3 cases were excluded due to missing follow-up data. *P* values were calculated using the log-rank test.
